# Chest Pain Network with Support of Telemedicine: Impact on Reperfusion Therapy and Clinical Outcomes After 8 Years of Experience

**DOI:** 10.1089/tmr.2021.0033

**Published:** 2021-12-22

**Authors:** Pedro Gabriel Melo de Barros e Silva, Thiago Andrade Macedo, Renato D. Lopes, Mariana Y. Okada, Tiago Frigini, Patricia O. Roveri, Rodrigo Balada, Lucas Silva de Macedo, Valter Furlan

**Affiliations:** ^1^ Hospital Samaritano Paulista, São Paulo, Brazil.; ^2^ Americas Serviços Medicos, São Paulo, Brazil.; ^3^ Centro Universitário São Camilo, São Paulo, Brazil.; ^4^ Duke Clinical Research Institute, Duke University Medical Center, Durham, North Carolina, USA.

**Keywords:** telemedicine, acute coronary syndrome, ST-segment elevation myocardial infarction

## Abstract

**Background:** Different approaches of evaluation by cardiologists using telemedicine have the potential of improving care of patients with ST elevation myocardial infarction (STEMI).

**Objective:** To compare the use of pharmacoinvasive strategy and associated clinical outcomes (heart failure [HF] and mortality) among patients with STEMI before and after a program of telemedicine and also according to the level of support by telemedicine.

**Methods:** A chest pain network with the support of a cardiologist through telemedicine was implemented in 2012 in 22 emergency departments without a local cardiac catheterization laboratory. Initially (phase 1 of telemedicine), the decision to discuss the case with the cardiologist was based on the judgment of the emergency physician. At the end of 2018, the use of telemedicine was modified and a dedicated cardiologist was available continuously to discuss systematically all suspected cases (phase 2 of telemedicine). The use of fibrinolytics and the rates of HF and in-hospital mortality were compared among three different periods: pretelemedicine (2011), and phase 1 and phase 2 of the telemedicine program.

**Results:** We evaluated 1034 STEMI patients and after comparing the three phases, we did not find significant differences regarding age, gender, and comorbidities. The use of fibrinolytics before transferring STEMI patients to a percutaneous coronary intervention center (pharmacoinvasive strategy) increased after telemedicine implementation (38% vs. 65.2%; *p* < 0.01), which was associated with a lower rate of HF (23.9% vs. 14.4%; *p* = 0.01) and death (7.9% vs. 4.0%; *p* = 0.05). The in-hospital mortality was lower in phase 2 with systematic evaluation by telemedicine compared with pretelemedicine (7.9% vs. 3.3%; *p* = 0.04).

**Conclusion:** The implementation of a systematic and organized chest pain protocol, including telemedicine support, was associated with a significant increase in the use of pharmacoinvasive strategy and better clinical patient outcomes in patients with STEMI. Our findings provide important insights on how to improve the management of this high-risk population, reducing the gap between evidence and clinical practice.

## Introduction

Registries of clinical practice that analyzed quality indicators of medical care among patients with acute coronary syndrome (ACS) have shown that there are gaps between evidence-based guideline recommendations and real-world medical practice.^1–4^ These gaps are even larger in low- and middle-income countries^3,4^ and, beyond the appropriate utilization of interventions with proved benefit, the timing of these interventions is also critical to achieve better clinical outcomes in time-sensitive conditions such as ST elevation myocardial infarction (STEMI).^5,6^ Different strategies have been tested to reduce these gaps, including training emergency department (ED) staff, and monitor quality indicators with regular feedback.^7–9^

These approaches have showed moderate benefit, but none of these represents a quality intervention that could be performed in real time during the diagnostic evaluation of patients with ACS.^7–9^ Telemedicine has the potential to check the quality metrics during the ED evaluation and help the decision-making process in the acute phase of time-sensitive conditions.^10–13^ Also, telemedicine coordination may help the decision regarding procedures such as percutaneous coronary intervention (PCI) and, consequently, decisions regarding hospital transfer of ACS patients.

Different approaches of evaluation by a cardiologist using telemedicine have been tested, including electrocardiographic transmissions, synchronous teleconsultations with a cardiologist, and direct physician/patient consultation.^11,12^ By different magnitudes, these interventions have the potential of improving care of patients with STEMI.^14^ Most of the evidence regarding the impact of telemedicine in patients with STEMI is related to prehospital electrocardiogram (ECG).^14^

An initial experience of using telemedicine for synchronous teleconsultation between emergency physicians and cardiologists has showed improvement in the use of reperfusion therapy, but there was a lack of information regarding the sustainability of this model of care and how to overcome the initial gaps that persisted in the first years of experience using telemedicine.^11^ In this synchronous teleconsultation model, the decision to contact the cardiologist depended on the judgment of the emergency physician. A model with a more comprehensive level of support, including systematic evaluation of all suspected cases, had not been assessed so far.

The objective of the current study is to compare the use of the fibrinolytics before transfer (pharmacoinvasive strategy) and clinical outcomes (heart failure [HF] and mortality) among patients admitted in non-PCI centers with STEMI, before and after a telemedicine program, and also according to the level of support offered by telemedicine during the 8 years of experience of the program.

## Methods

### Study design

A retrospective cohort of patients with STEMI who received initial care in a non-PCI center in a chest pain network of private EDs. The study was approved by the Institutional Review Board.

### Hub and spoke network

A chest pain network based on a spoke and hub model with the support of a cardiologist through telemedicine was implemented in 2012 with 22 hospitals and EDs.^11^ Since 2012, there were at least two cardiologists on call 24 h a day, 7 days a week (both capable of discussing cardiologic cases by telemedicine). In the first phase of the program (from 2012 to the end of 2018), the cardiologists did not work exclusively in telemedicine, but were also responsible for the medical care of cardiac patients in the ED of the cardiologic hub.

In the second phase of the program, a cardiologist was dedicated exclusively to assisting other hospitals by telemedicine, while the second was responsible for medical care in the emergency room and would only give support by telemedicine in cases of simultaneous calls (contingency). All the STEMI cases were transferred to a PCI center (hub) from the same private group during the entire period of analysis (from 2011 to 2019). All the sites are located in the metropolitan region of Sao Paulo. The EDs in the spoke sites are not specialized in cardiology, while the ED in the hub had cardiologists on-site 24 h/day, 7 days/week.

During the entire period of analysis (before and during the telemedicine program), the protocol for STEMI treatment was the same (aspirin, clopidogrel, and enoxaparin) and the recommendation was a pharmacoinvasive approach as the standard reperfusion therapy for all eligible patients in non-PCI centers. The option of pharmacoinvasive as a default for reperfusion in spoke centers was based on the fact that ambulance services varied according to the health plan and there was no clear service level agreement that could guarantee a transportation fast enough for primary PCI. Nevertheless, the results of pharmacoinvasive treatment showed that this approach is a good alternative to primary PCI, especially in settings where the primary PCI is not certain.^15–17^

### Telemedicine program

In 2011, the cases were transferred without previous discussion regarding treatment options and all the decisions related to acute medical care were made by the attending physician in the ED of a non-PCI center.

Since 2012 (year of implementation), the attending physicians from the ED of a non-PCI center (spoke) could contact a cardiologist from the hub to support medical decision before transferring the patient. Initially (phase 1 of telemedicine), the decision to discuss the case with the cardiologist was based on the judgment of the emergency physician in each facility (spoke). In 2018, the use of telemedicine was modified and a dedicated cardiologist was available continuously to discuss systematically all suspected cases (phase 2 of telemedicine).

The telemedicine program included a system of videoconference and evaluation of the ECG with Zoom feature. We used a teleconferencing system that allows transmission in high definition (Polycom), which facilitates the discussion of clinical cases. The evaluation of the ECG was both by a projector with Zoom feature (through the projector's Elmo^®^ [Westminster, CA] document camera) and an internal internet-based electronic form for ECG upload. All services were performed by a high-speed connection with at least 4 MB dedicated for each link (>100 MB for the network) during both phases of the program. The contingency for cases of technical problems in the videoconference platform was the use of telephone for clinical discussion.

The telemedicine cardiologist had the role to support medical decision and activated the system for medical transportation in cases that required more complex diagnostic or therapeutic resources (all STEMI and unstable cases were routinely transferred to a hub). The data related to each consultation via telemedicine were collected using a standardized form filled out by the telemedicine physicians and these data were maintained in a central database. The data of all cases transferred to the hub due to STEMI were included in the chest pain protocol databank of the referral hospital.

**FIG. 1.**
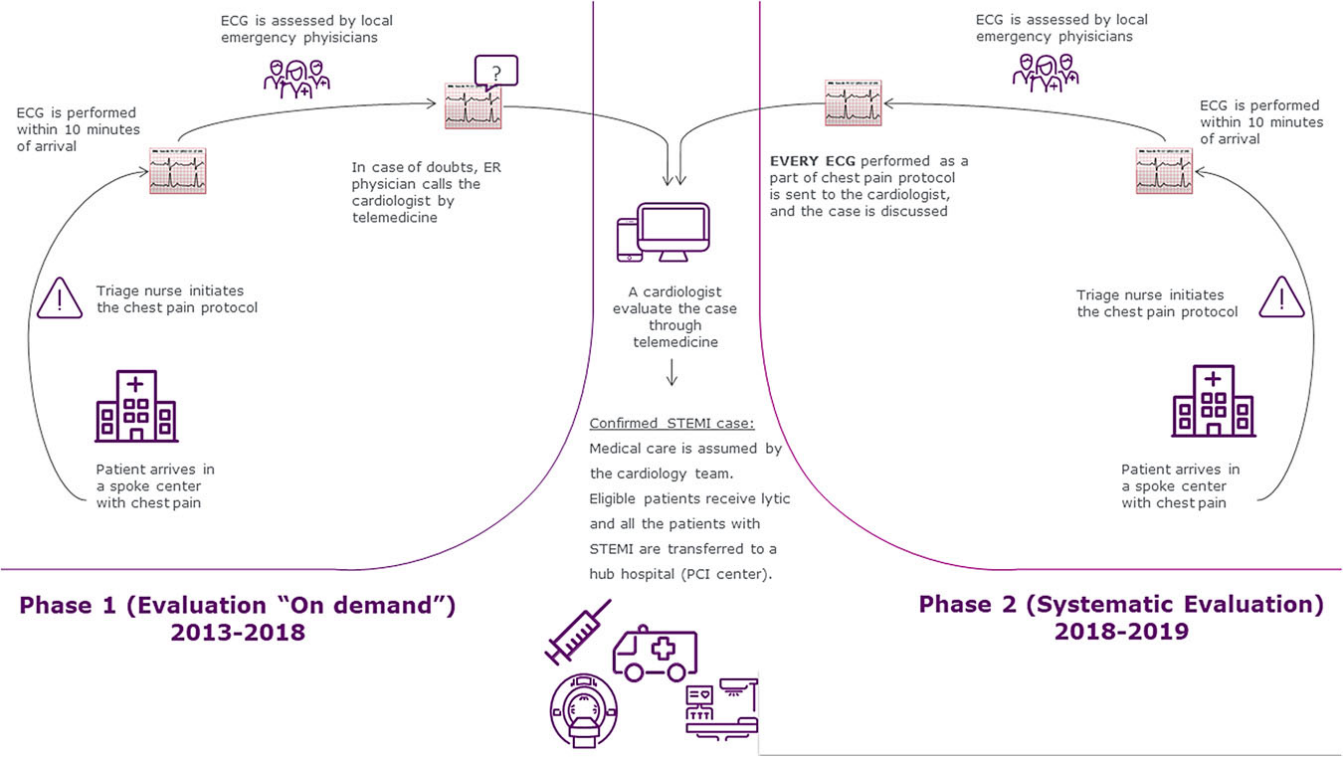
Summary of the telemedicine program (phases 1 and 2).

A summary of the telemedicine program is shown in Figure 1.

### Goals of the chest pain network (end-points of the current analysis)

The main goals of this network were to improve the quality of care in the initial assessment and also guide the rational use of resources (avoid unnecessary transfers, selecting the most complex patients to the appropriate facility). Quality metrics were continuously measured since 2011 (pretelemedicine) and included the use of fibrinolytics and event rate of HF and in-hospital mortality. These measures are the end-points of the current analysis that compared these metrics in three different periods: pretelemedicine, and phase 1 and phase 2 of the telemedicine program. HF was considered all the cases of ejection fraction <40% and/or signs of HF during STEMI hospitalization. Patients who met these criteria without previous diagnosis of HF were considered “new HF.”

### Study participants

All consecutive patients transferred from a non-PCI center (spoke) to the hub of a chest pain network due to a confirmed diagnosis of STEMI from January 2011 to December 2019 were included in the current analysis. The reference hospital in the current analysis is a private general hospital focused on cardiovascular diseases with international quality accreditation and which has been using the National Cardiovascular Data Registries (NCDR^®^) as a quality improvement tool since 2012.

### Statistical analyses

Categorical variables were reported by the absolute and relative frequencies, and continuous variables were described by mean and standard deviation. Comparisons between groups were made using *t*-test for continuous variables and chi-square for categorical variables. *p* Values were two-tailed, and values below 0.05 were considered statistically significant. The two clinical outcomes (in-hospital mortality and HF at hospital discharge) were also included in a logistic regression multivariate analysis to evaluate the independent association of these indicators with the period of telemedicine (2011 vs. 2013–2019).

The results were reported as odds ratio (OR) and the following baseline covariates were included in this analysis: age, gender, and comorbidities (hypertension, dyslipidemia, diabetes, smoking, previous MI, and previous HF). All analyses were performed using R software, version 3.6.1 (R Foundation for Statistical Computing).

## Results

### Baseline characteristics

We evaluated 1034 STEMI patients from 2011 to 2019 (113 pretelemedicine; 645 in phase 1; and 276 in phase 2). In the overall population included in the analysis, 75.8% were male patients with a mean age of 58.5 ± 11 years. The most common risk factor was arterial hypertension (62.1%) and 8.4% had a history of myocardial infarction. Comparing the three phases, we did not find differences regarding age, gender, and comorbidities (Table 1).

**Table 1.** Clinical Characteristics of the Transferred Patients Before and After Telemedicine (Phase 1 and Phase 2)

	**Pretelemedicine (2011)** ***n* = 113**	**Phase 1 (2013–2018)** ***n* = 645**	**Phase 2 (2018–2019)** ***n* = 276**	***p***
Age in years (SD)	59.6 ± 13	58.1 ± 10	59.2 ± 12	0.16 and 0.77
Male (%)	79/113 (69.9)	490/645 (75.9)	215/276 (77.8)	0.16 and 0.09
Hypertension (%)	78/113 (69.0)	399/645 (61.8)	166/276 (60.1)	0.14 and 0.10
Dyslipidemia (%)	52/113 (46.0)	264/645 (40.9)	102/276 (36.9)	0.31 and 0.09
Diabetes (%)	31/113 (27.4)	142/645 (22.0)	55/276 (19.9)	0.20 and 0.10
Smoking (%)	35/113 (30.9)	232/645 (35.9)	105/276 (38.0)	0.30 and 0.18
Previous MI (%)	8/113 (7.0)	53/645 (8.2)	26/276 (9.4)	0.68 and 0.45
Previous HF (%)	7/113 (6.1)	25/645 (3.8)	9/276 (3.2)	0.25 and 0.18
Killip classification of ≥2 (%)	25/113 (22.1)	85/645 (13.1)	25/276 (9.0)	0.01 and <0.01

HF, heart failure; MI, myocardial infarction; SD, standard deviation.

### Comparison pre- and post-telemedicine

**FIG. 2.**
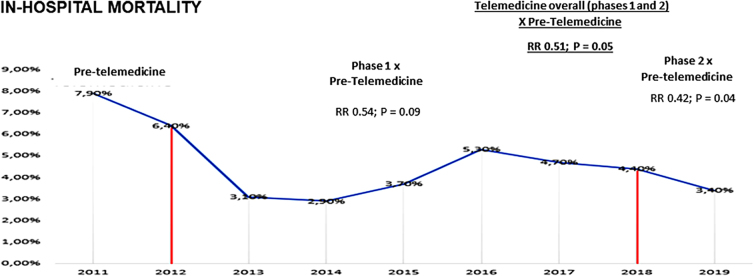
In-hospital mortality of STEMI patients during the 8 years of telemedicine program (phases 1 and 2). STEMI, ST elevation myocardial infarction.

The use of fibrinolytics before transferring STEMI patients to a PCI center (pharmacoinvasive strategy) increased by 71% after telemedicine implementation (38% vs. 65.2%; *p* < 0.01) (Table 2). This improvement in quality of care was associated with a 41% lower rate of HF during hospitalization (23.9% vs. 14.4%; *p* < 0.01 for overall; 18.8% vs. 11.1% for new HF) compared with the period pretelemedicine (Table 2). There was also an absolute 5.3% higher ejection fraction in the period post-telemedicine compared with the period pretelemedicine (Table 2). A trend of lower in-hospital mortality was identified in the same period (7.9% vs. 4.0%; *p* = 0.05) (Table 2 and Fig. 2).

**Table 2.** Study End-Points Before and After Telemedicine (Average of Phase 1 and Phase 2)

	**Pretelemedicine (2011)**	**Telemedicine (2013–2019)**	***p***
STEMI cases/year	113	131.5	—
Fibrinolytics (%)	43/113 (38.0)	601/921 (65.2)	<0.01
In-hospital mortality (%)	9/113 (7.9)	37/921 (4.0)	0.05
Ejection fraction (SD)	49.9% (±15.3%)	55.2% (±12.4%)	<0.01
HF (%)	27/113 (23.9)	133/921 (14.4)	0.01
New HF (%)	20/106 (18.8)	99/887 (11.1)	0.02

STEMI, ST elevation myocardial infarction.

### Comparison between pretelemedicine and phases 1 and 2

The use of fibrinolytics was higher in the phase 2 of the telemedicine program compared not only with the pretelemedicine phase (38% vs. 85.1%; *p* < 0.01) but also compared with the phase 1 of the telemedicine program (58.1% vs. 85.1%; *p* < 0.01). The rate of total HF and HF was not statistically different between phases 1 and 2, but both phases showed a reduction around 41% compared with pretelemedicine (Table 3). Regarding in-hospital mortality, there was a numerical reduction in both phases compared with pretelemedicine, but only in phase 2 the reduction was statistically significant (7.9% vs. 3.3%; *p* = 0.04) (Table 2).

**Table 3.** Comparison Between Pretelemedicine and the Two Phases of Implementation

	**Pretelemedicine (2011)** ***n* = 113**	**Phase 1 (2013–2018)** ***n* = 645**	**Phase 2 (2018–2019)** ***n* = 276**	***p*** ^*^
Fibrinolytics (%)	43/113 (38.0)	375/645 (58.1%)	235/276 (85.1%)	<0.01 (both)
Mortality (%)	9/113 (7.9)	28/645 (4.3%)	9/276 (3.3%)	0.09 and 0.04
HF (%)	27/113 (23.9)	94/645 (14.5%)	39/276 (14.1%)	0.01 (both)
New HF (%)	20/106 (18.8)	69/620 (11.1%)	30/268 (11.1%)	0.02 and 0.04

^*^ *p*-Value of comparison between pretelemedicine and phase 1 and between pretelemedicine and phase 2.

### Multivariate analysis

A multivariate analysis showed that controlling for baseline variables there was an independent association with lower in-hospital mortality (OR = 0.54; confidence interval [CI] 95% 0.22–0.87; *p* < 0.01) and lower rate of HF at hospital discharge (OR = 0.62; CI 95% 0.30–0.93; *p* < 0.01) during the telemedicine program when compared with the period pretelemedicine. When excluding the period pretelemedicine and analyzing only during the years of telemedicine program (2013–2019) separating groups in phase 1 and phase 2 periods, the ORs of in-hospital mortality and rate of HF during phase 2 were not statistically significant in the multivariate analysis.

## Discussion

The current study evaluated the use of reperfusion therapy and clinical outcomes among patients initially evaluated in non-PCI centers from a hub and spoke chest pain network. The patients were categorized in groups according to the level of support by telemedicine.

The analysis showed that the use of telemedicine was associated with improvement in the pharmacoinvasive strategy and a lower rate of in-hospital HF and mortality, including a multivariate analysis that showed an independent association of better clinical outcomes with the period of telemedicine program. Despite the absence of difference of clinical outcomes between the two phases of the program in the multivariate analysis, the increment of use of reperfusion therapy was maximum during the second phase, with a systematic support of telemedicine in all cases included in the chest pain protocol.

The potential benefit of telemedicine in increasing reperfusion therapy was assessed in previous studies, including a report of the initial experience of the chest pain network evaluated in the current analysis.^11^ This preliminary report explored the first 2 years after implementation of phase 1 of the telemedicine program and did not include the following 3.5 years of phase 1 and the 1.5 years of phase 2. This initial analysis of a short period after implementation of a telemedicine program is limited due to the possibility of nonsustainability of the initial findings.

Even assuming sustainability of the results, the improvements in the procedures related to telemedicine support would not be captured in the first phase of implementation since a quality improvement process needs continuous evaluations and interventions.^18–20^ Also, the lower number of patients and events limited the statistical power to assess meaningful changes in clinical outcomes beyond the initial improvement in quality metrics related to evidence-based therapies.

Telemedicine is one of the tools that could reduce the gap between evidence-based guideline recommendation and real-world medical practice.^11–13^ In the current analysis, this tool was not used alone, but all the medical and nurse staff from the EDs of the non-PCI centers also received training of the chest pain protocol and they also monitored their results with monthly feedback.

Thus, the results of the current analysis should be considered in a scenario were telemedicine is offered in a multifaceted quality improvement intervention and not alone. It is important to take into account that even with well-conducted quality improvement interventions based on quality metrics, residual gaps related to adherence of evidence-based therapies are commonly observed in STEMI patients.^8,12^ This could occur because most of the quality improvement interventions are retrospective but telemedicine adds a real-time intervention, which has a great potential of benefit in time-sensitive scenarios (such as STEMI) where the physician could be insecure regarding diagnosis (STEMI or not) and treatment (e.g., use of lytics).

Regarding the different approaches used by telemedicine, despite the initial improvement identified in phase 1 of the program, gaps were still identified especially because the non-PCI center physician could not identify the STEMI or had the choice of not discussing the case by telemedicine. These gaps were more commonly identified after the first 2 years of the telemedicine program, which led to a decline in the use of fibrinolytics at the end of phase 1. Thus, the systematic approach was implemented in phase 2 to reach these cases not discussed by telemedicine and enhance the coverage of the service recommending the discussion of all patients included in a chest pain protocol.

The improvements of the targets in the telemedicine program were clearly identifiable, with the highest rate of reperfusion therapy before transfer (pharmacoinvasive strategy) in the year of 2019, which reinforces the importance of systematic discussion by telemedicine. This enhance in pharmacoinvasive strategy was associated with better clinical outcomes, including a statistically significant lower mortality in the phase 2 compared with the period pretelemedicine.

The current STEMI guidelines do not address specific recommendations regarding telemedicine, but they recommend prehospital ECG, especially for patients who received emergency medical service (EMS) support by ambulance,^15–17^ since most of the evidence is related to this type of support.^14^ Nevertheless, in many countries, a patient commonly seeks help in non-PCI EDs as a walk-in, without EMS activation and this situation limits the approach based on prehospital ECG. Beyond the low utilization of EMS for patients with STEMI, in countries such as Brazil, emergency physicians commonly did not receive formal training in emergency care and have diverse medical specialties.

In this scenario, telemedicine has more potential to enhance adherence to evidence-based therapies and improve clinical outcomes. Thus, the continuous support of a cardiologist would have a higher impact on services that have more variation regarding the experience of the emergency physician. This implementation should be in a spoke and hub network^21,22^ with homogeneous protocol and ideally associated with continuous training of the emergency physicians (including training on how to use telemedicine) and continuous monitoring of quality indicators with regular feedback. The continuous quality improvement initiatives are essential since telemedicine needs to improve their processes along the time as occurred in the phase 2 of the current study.

Despite the absence of more broad recommendations in STEMI guidelines, documents to guide telemedicine use include STEMI patients as a group with potential for improving medical care using telemedicine, based on limited evidence.^23^

### Study limitations

Since this was not a randomized study, differences among the groups could explain the differences in outcomes. One factor that minimizes this issue is the fact that the demographic variables and comorbidities analyzed were not different among the three groups. Also, the end-point of adherence to thrombolysis before transfer would not be influenced by baseline characteristics since all the patients had STEMI. Regarding medical factors beyond telemedicine that could influence the results, it is important to reinforce that this analysis was performed in the same chest pain network and the therapeutic scheme recommended by the medical protocol with dual antiplatelet therapy, anticoagulation, and reperfusion therapy did not change along the years.

In addition, the changes from pretelemedicine to the showed phase 1 and also from phase 1 to phase 2 showed a clear temporal relationship between intervention and results. Finally, considering external validity, countries or hospitals that already achieved the quality targets using protocols with trained emergency physicians and regular monitoring of quality indicators may not have the same benefit with the addition of telemedicine, which should be considered a useful tool for services that still had gaps despite the local resources.

Also, the use of the approach presented in the current study may be limited due to legal restrictions in different countries^23,24^ despite greater flexibility during the pandemic of COVID-19.

## Conclusion

In an 8-year analysis, the implementation of a chest pain protocol including telemedicine support was associated with a significant increase in the use of pharmacoinvasive strategy and a lower rate of death and HF in patients with STEMI. Our findings provide important insights on how to improve the management of this high-risk population, reducing the gap between evidence and clinical practice.

## Abbreviations Used

ACS: acute coronary syndrome
CI: confidence interval
ECG: electrocardiogram
ED: emergency department
EMS: emergency medical service
HF: heart failure
MI: myocardial infarction
NCDR^®^: National Cardiovascular Data Registry
OR: odds ratio
PCI: percutaneous coronary intervention
SD: standard deviation
STEMI: ST elevation myocardial infarction
